# Supplementary material for: Exploring differences between gender expressions in exercise self-efficacy and outcome expectations for exercise in individuals with stroke
Source: PLoS One. 2024 Mar 13;19(3):e0299288. doi: 10.1371/journal.pone.0299288 (PMC10936775; doi:10.1371/journal.pone.0299288)
Supplement: S1 Table — (DOCX) [file pone.0299288.s001.docx]

| **S1 Table. Characteristics of the outliers removed from the model exploring the differences in gender expression and outcome expectations for exercise** | | | | | | | | |
| --- | --- | --- | --- | --- | --- | --- | --- | --- |
|  | **Gender Expression Group** | **SOEE score** | **Sex** | **Age** | **Time post-stroke (years)** | **Stroke Type** | **Affected Limb** | **mRS Score** |
| Participant #1 | Masculine | 2.2 | Male | 59 | 3.3 | Ischemic | Left | 2 |
| Participant #2 | Undifferentiated | 1.8 | Female | 78 | 4.2 | Unknown | Left | 4 |
| Participant #3 | Androgynous | 2.6 | Male | 69 | 4.2 | Ischemic | Right | 3 |
| *Note.* SOEE= Short Outcome Expectations for Exercise; mRS= Modified Rankin Scale | | | | | | | | |
